# Aqua­(4-nitro­phthalato)bis­[2-(1*H*-pyrazol-3-yl)pyridine]­zinc(II) hemihydrate

**DOI:** 10.1107/S1600536810051949

**Published:** 2010-12-24

**Authors:** Lei Ni, Ji-Li Zhao, Hong Wei

**Affiliations:** a College of Chemistry and Biology, Beihua University, Jilin 132013, People’s Republic of China

## Abstract

In the title compound, [Zn(C_8_H_3_NO_6_)(C_8_H_7_N_3_)_2_(H_2_O)]·0.5H_2_O, the Zn^II^ atom shows a distorted octa­hedral ZnN_4_O_2_ coordination environment and is bonded to two 3-(2-pyrid­yl)-1*H*-pyrazole ligands *via* the N atoms, one monodentate 4-nitro­phthalate ligand and one associated water mol­ecule. Additionally, one water of crystallization, with a site-occupation factor of 0.5, is present. The two 3-(2-pyrid­yl)-1*H*-pyrazole ligands are planar [r.m.s. deviations = 0.03 (1) and 0.35 (1) Å] and the dihedral angle between the two planar 3-(2-pyrid­yl)-1*H*-pyrazole ligands is 67.31 (4)°. Inter­molecular π–π stacking inter­actions between 3-(2-pyrid­yl)-1*H*-pyrazole ligands with a face-to-face separation of 3.64 (1) Å are observed. Moreover, the crystal structure is stabilized by O—H⋯O and N—H⋯O hydrogen bonds between the water of crystallization, the associated water mol­ecule and the 3-(2-pyrid­yl)-1*H*-pyrazole ligands.

## Related literature

For background to metal-organic frameworks, see: Hagrman *et al.* (1999 ▶); Kitagawa *et al.* (2004 ▶).
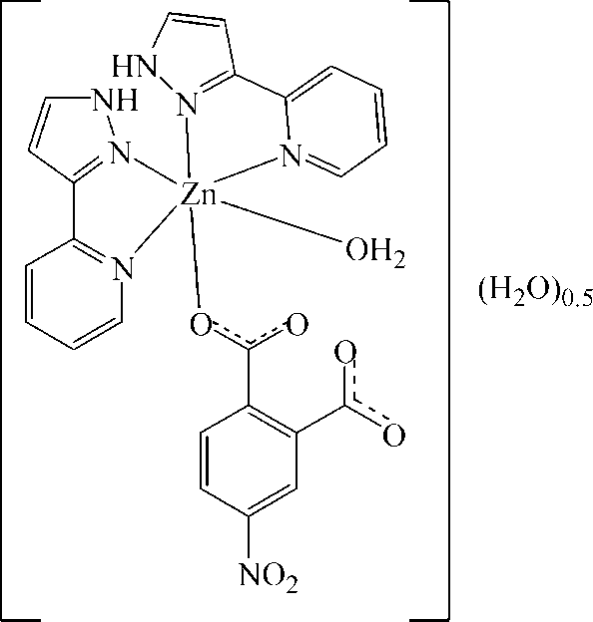


## Experimental

### 

#### Crystal data

[Zn(C_8_H_3_NO_6_)(C_8_H_7_N_3_)_2_(H_2_O)]·0.5H_2_O
*M* *_r_* = 591.84
Triclinic, 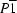

*a* = 10.4284 (6) Å
*b* = 11.1844 (7) Å
*c* = 11.9656 (8) Å
α = 96.508 (3)°
β = 112.091 (3)°
γ = 96.366 (3)°
*V* = 1266.84 (14) Å^3^
*Z* = 2
Mo *K*α radiation
μ = 1.03 mm^−1^
*T* = 294 K
0.12 × 0.10 × 0.08 mm

#### Data collection

Bruker APEXII CCD diffractometer
Absorption correction: multi-scan (*SADABS*; Bruker, 2001 ▶) *T*_min_ = 0.886, *T*_max_ = 0.922
9062 measured reflections
4258 independent reflections
3825 reflections with *I* > 2σ(*I*)
*R*_int_ = 0.020

#### Refinement

*R*[*F* ^2^ > 2σ(*F* ^2^)] = 0.035
*wR*(*F* ^2^) = 0.104
*S* = 1.00
4258 reflections
364 parameters
3 restraints
H atoms treated by a mixture of independent and constrained refinement
Δρ_max_ = 0.65 e Å^−3^
Δρ_min_ = −0.42 e Å^−3^

### 

Data collection: *APEX2* (Bruker, 2004 ▶); cell refinement: *SAINT-Plus* (Bruker, 2001 ▶); data reduction: *SAINT-Plus*; program(s) used to solve structure: *SHELXS97* (Sheldrick, 2008 ▶); program(s) used to refine structure: *SHELXL97* (Sheldrick, 2008 ▶); molecular graphics: *SHELXTL* (Sheldrick, 2008 ▶); software used to prepare material for publication: *SHELXTL*.

## Supplementary Material

Crystal structure: contains datablocks I, global. DOI: 10.1107/S1600536810051949/im2226sup1.cif

Structure factors: contains datablocks I. DOI: 10.1107/S1600536810051949/im2226Isup2.hkl

Additional supplementary materials:  crystallographic information; 3D view; checkCIF report

## Figures and Tables

**Table 1:** Hydrogen-bond geometry (Å, °)

*D*—H⋯*A*	*D*—H	H⋯*A*	*D*⋯*A*	*D*—H⋯*A*
O1—H2*W*⋯O5^i^	0.82 (3)	1.81 (3)	2.627 (3)	174 (3)
N5—H5*A*⋯O4^i^	0.86	1.95	2.788 (3)	166
N2—H2*A*⋯O2	0.86	1.82	2.606 (4)	150

Symmetry code: (i) 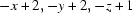.

**Fig. 1.**
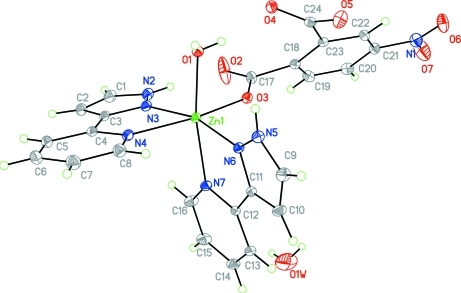
Molecular structure of the title compound with the atom-labelling scheme. Displacement ellipsoids are drawn at the 30% probability level; H atoms are given as spheres of arbitrary radius.

**Fig. 2.**
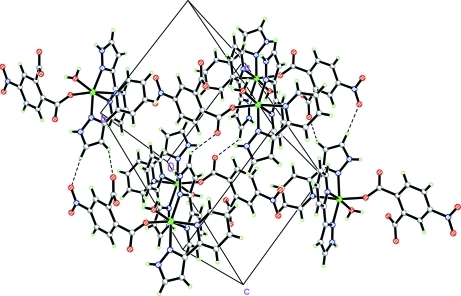
Crystal packing of the title compound, displayed with hydrogen bonds as dashed lines.

## supplementary crystallographic information

### Comment

The synthesis of entangled metal-organic frameworks (MOFs) has attracted continuous research interest not only because of their appealing structural and topological novelty, but also due to their unusual optical, electronic, magnetic, and catalytic properties, as well as their potential medical application (Hagrman *et al.* (1999); Kitagawa *et al.* (2004)). Here, we describe the synthesis and structural characterization of the title compound resulting from an attempted MOF synthesis in which Zn and Gd complex building blocks were expected to be the constituents.

Single crystal X-ray diffraction analyses revealed that the asymmetric unit of the title compound, [(Zn(C_8_H_7_N_3_)_2_(C_8_NH_3_O_6_)(H_2_O)].(H_2_O)_0.5_, consists of one Zn^2+^ ion, two 3-(2-pyridyl)-1*H*-pyrazole ligands, one 4-Nitrophthalato ligand, one associated water molecule, and half water of crystallization. As shown in Figure 1, the Zn^2+^ ion is hexacoordinated by four N atoms from 3-(2-pyridyl)-1*H*-pyrazole ligands and two oxygen atoms from 4-Nitrophthalato ligand and one associated water molecule, exhibiting a distorted octahedral arrangement of ZnN_4_O_2_. Moreover, the Rms deviations of the two 3-(2-pyridyl)-1*H*-pyrazole groups from planarity are 0.03 (1) and 0.35 (1) Å, respectively. The dihedral angle between the two 3-(2-pyridyl)-1*H*-pyrazole planars is 67.31 (4) Å. Interestingly, between the molecules, there is π-π stacking interactions with the face-to-face separation (*ca* 3.64 (1) Å) between the 3-(2-pyridyl)-1*H*-pyrazole planars. Meantime, there are extensive hydrogen bonds between water of crystallization, associated water molecule, and 3-(2-pyridyl)-1*H*-pyrazole, and leads to a consolidation of the structure (Figure 2).

### Experimental

A mixture of zinc oxalate dihydrate (0.26 mmol, 0.050 g), 3-(2-pyridyl)-1*H*-pyrazole (0.32 mmoL, 0.05 g), 4-nitrophthalic acid (0.24 mmoL, 0.05 g), gadolinium(III) nitrate pentahydrate (0.12 mmoL, 0.05 g), and 14 ml H_2_O was sealed in a 25 ml Teflon-lined stainless steel autoclave at 433 K for three days. Pink crystals suitable for the X-ray experiment were obtained. The yield is 60% based on the zinc salt. Anal. Calc. for C_48_H_40_N_14_O_15_Zn_2_: C 48.65, H 3.38, N 16.55%; Found: C 48.32, H 3.15, N 16.39%.

### Refinement

All hydrogen atoms bound to carbon were refined using a riding model with distance C—H = 0.93 Å, *U*_iso_ = 1.2*U*_eq_ (C) for aromatic atoms. The H atoms of the coordinated water molecule were located from difference density maps and were refined with d(O—H) = 0.83 (2) Å, and with a fixed *U*_iso_ of 0.80 Å^2^. H atoms at the solvent water molecule could not be derived from the Fourier map. Due to the site occupation factor of 0.5 for O1W these positions wer excluded from the final refinement.

### Crystal data

**Table:**

[Zn(C_8_H_3_NO_6_)(C_8_H_7_N_3_)_2_(H_2_O)]·0.5H_2_O	*Z* = 2
*M**_r_* = 591.84	*F*(000) = 604
Triclinic, *P*1	*D*_x_ = 1.552 Mg m^−^^3^
Hall symbol: -P 1	Mo *K*α radiation, λ = 0.71073 Å
*a* = 10.4284 (6) Å	Cell parameters from 4528 reflections
*b* = 11.1844 (7) Å	θ = 2.4–27.3°
*c* = 11.9656 (8) Å	µ = 1.03 mm^−^^1^
α = 96.508 (3)°	*T* = 294 K
β = 112.091 (3)°	Block, colorless
γ = 96.366 (3)°	0.12 × 0.10 × 0.08 mm
*V* = 1266.84 (14) Å^3^	

### Data collection

**Table:**

Bruker APEXII CCD diffractometer	4258 independent reflections
Radiation source: fine-focus sealed tube	3825 reflections with *I* > 2σ(*I*)
graphite	*R*_int_ = 0.020
phi and ω scans	θ_max_ = 25.0°, θ_min_ = 1.9°
Absorption correction: multi-scan (*SADABS*; Bruker, 2001)	*h* = −12→12
*T*_min_ = 0.886, *T*_max_ = 0.922	*k* = −13→13
9062 measured reflections	*l* = −14→13

### Refinement

**Table:**

Refinement on *F*^2^	Primary atom site location: structure-invariant direct methods
Least-squares matrix: full	Secondary atom site location: difference Fourier map
*R*[*F*^2^ > 2σ(*F*^2^)] = 0.035	Hydrogen site location: inferred from neighbouring sites
*wR*(*F*^2^) = 0.104	H atoms treated by a mixture of independent and constrained refinement
*S* = 1.00	*w* = 1/[σ^2^(*F*_o_^2^) + (0.066*P*)^2^ + 0.7068*P*] where *P* = (*F*_o_^2^ + 2*F*_c_^2^)/3
4258 reflections	(Δ/σ)_max_ = 0.001
364 parameters	Δρ_max_ = 0.65 e Å^−^^3^
3 restraints	Δρ_min_ = −0.42 e Å^−^^3^

### Special details

**Table:**

Geometry. All e.s.d.'s (except the e.s.d. in the dihedral angle between two l.s. planes) are estimated using the full covariance matrix. The cell e.s.d.'s are taken into account individually in the estimation of e.s.d.'s in distances, angles and torsion angles; correlations between e.s.d.'s in cell parameters are only used when they are defined by crystal symmetry. An approximate (isotropic) treatment of cell e.s.d.'s is used for estimating e.s.d.'s involving l.s. planes.
Refinement. Refinement of *F*^2^ against ALL reflections. The weighted *R*-factor *wR* and goodness of fit *S* are based on *F*^2^, conventional *R*-factors *R* are based on *F*, with *F* set to zero for negative *F*^2^. The threshold expression of *F*^2^ > σ(*F*^2^) is used only for calculating *R*-factors(gt) *etc*. and is not relevant to the choice of reflections for refinement. *R*-factors based on *F*^2^ are statistically about twice as large as those based on *F*, and *R*- factors based on ALL data will be even larger.

### Fractional atomic coordinates and isotropic or equivalent isotropic displacement parameters (Å^2^)

**Table:**

	*x*	*y*	*z*	*U*_iso_*/*U*_eq_	
C1	0.7155 (4)	0.7772 (3)	0.9195 (3)	0.0652 (9)	
H1	0.6311	0.7613	0.9291	0.078*	
C2	0.8440 (4)	0.8158 (3)	1.0110 (3)	0.0618 (9)	
H2	0.8654	0.8318	1.0946	0.074*	
C3	0.9371 (3)	0.8262 (2)	0.9526 (3)	0.0471 (7)	
C4	1.0889 (3)	0.8628 (2)	0.9968 (3)	0.0476 (7)	
C5	1.1747 (4)	0.9022 (3)	1.1197 (3)	0.0610 (8)	
H5	1.1367	0.9060	1.1788	0.073*	
C6	1.3160 (4)	0.9354 (3)	1.1522 (3)	0.0719 (10)	
H6	1.3755	0.9618	1.2339	0.086*	
C7	1.3695 (4)	0.9292 (3)	1.0618 (3)	0.0708 (10)	
H7	1.4651	0.9517	1.0817	0.085*	
C8	1.2775 (4)	0.8889 (3)	0.9417 (3)	0.0589 (8)	
H8	1.3136	0.8841	0.8814	0.071*	
C9	1.2913 (3)	0.7454 (3)	0.5661 (3)	0.0594 (8)	
H9	1.3580	0.7702	0.5356	0.071*	
C10	1.2595 (3)	0.6312 (3)	0.5882 (3)	0.0576 (8)	
H10	1.2987	0.5629	0.5755	0.069*	
C11	1.1556 (3)	0.6392 (2)	0.6339 (2)	0.0381 (5)	
C12	1.0801 (3)	0.5491 (2)	0.6763 (2)	0.0369 (5)	
C13	1.0886 (3)	0.4254 (2)	0.6620 (3)	0.0493 (7)	
H13	1.1427	0.3953	0.6223	0.059*	
C14	1.0159 (4)	0.3486 (3)	0.7075 (3)	0.0621 (9)	
H14	1.0200	0.2656	0.6988	0.075*	
C15	0.9378 (4)	0.3948 (3)	0.7654 (4)	0.0680 (9)	
H15	0.8888	0.3442	0.7977	0.082*	
C16	0.9322 (4)	0.5181 (3)	0.7755 (3)	0.0606 (8)	
H16	0.8783	0.5491	0.8150	0.073*	
C17	0.6685 (3)	0.7186 (3)	0.5137 (3)	0.0499 (7)	
C18	0.5894 (3)	0.6933 (2)	0.3769 (2)	0.0403 (6)	
C19	0.4792 (3)	0.5955 (3)	0.3277 (3)	0.0539 (7)	
H19	0.4518	0.5534	0.3799	0.065*	
C20	0.4097 (3)	0.5596 (3)	0.2034 (3)	0.0586 (8)	
H20	0.3366	0.4936	0.1711	0.070*	
C21	0.4512 (3)	0.6235 (3)	0.1282 (3)	0.0496 (7)	
C22	0.5606 (3)	0.7203 (2)	0.1733 (2)	0.0431 (6)	
H22	0.5887	0.7600	0.1200	0.052*	
C23	0.6285 (2)	0.7579 (2)	0.2984 (2)	0.0371 (5)	
C24	0.7389 (3)	0.8724 (2)	0.3428 (3)	0.0442 (6)	
Zn1	0.97417 (3)	0.78410 (2)	0.71551 (3)	0.03624 (12)	
N1	0.3763 (3)	0.5867 (3)	−0.0053 (3)	0.0720 (8)	
N2	0.7314 (3)	0.7660 (2)	0.8127 (2)	0.0519 (6)	
H2A	0.6641	0.7428	0.7420	0.062*	
N3	0.8669 (3)	0.7961 (2)	0.8318 (2)	0.0452 (5)	
N4	1.1400 (3)	0.8564 (2)	0.9082 (2)	0.0477 (6)	
N5	1.2091 (2)	0.8153 (2)	0.5964 (2)	0.0442 (5)	
H5A	1.2100	0.8911	0.5895	0.053*	
N6	1.1256 (2)	0.75232 (18)	0.63868 (19)	0.0374 (5)	
N7	1.0006 (2)	0.59453 (19)	0.7312 (2)	0.0423 (5)	
O1	0.95913 (19)	0.95872 (16)	0.67148 (17)	0.0420 (4)	
O2	0.5984 (3)	0.7250 (6)	0.5755 (3)	0.165 (2)	
O3	0.79649 (18)	0.72281 (17)	0.55259 (17)	0.0433 (4)	
O4	0.7478 (3)	0.94256 (18)	0.43546 (19)	0.0594 (6)	
O5	0.8072 (3)	0.8910 (2)	0.2798 (3)	0.0751 (7)	
O6	0.4156 (3)	0.6389 (3)	−0.0729 (2)	0.0957 (10)	
O7	0.2773 (4)	0.5050 (4)	−0.0427 (3)	0.1448 (18)	
O1W	0.5000	0.0000	0.5000	0.198 (4)	
H1W	0.901 (2)	0.943 (3)	0.6007 (10)	0.080*	
H2W	1.031 (2)	1.004 (3)	0.681 (3)	0.080*	

### Atomic displacement parameters (Å^2^)

**Table:**

	*U*^11^	*U*^22^	*U*^33^	*U*^12^	*U*^13^	*U*^23^
C1	0.086 (3)	0.066 (2)	0.074 (2)	0.0190 (18)	0.061 (2)	0.0210 (17)
C2	0.092 (3)	0.0636 (19)	0.0523 (18)	0.0227 (18)	0.0484 (19)	0.0194 (15)
C3	0.0754 (19)	0.0368 (13)	0.0434 (15)	0.0165 (13)	0.0361 (14)	0.0114 (11)
C4	0.0715 (19)	0.0345 (13)	0.0434 (15)	0.0159 (13)	0.0270 (14)	0.0103 (11)
C5	0.091 (3)	0.0509 (17)	0.0440 (16)	0.0205 (17)	0.0269 (17)	0.0116 (13)
C6	0.088 (3)	0.062 (2)	0.0500 (19)	0.0151 (18)	0.0103 (18)	0.0082 (15)
C7	0.066 (2)	0.065 (2)	0.069 (2)	0.0092 (17)	0.0149 (18)	0.0103 (17)
C8	0.0617 (19)	0.0566 (18)	0.0591 (19)	0.0092 (15)	0.0251 (16)	0.0092 (15)
C9	0.0543 (18)	0.072 (2)	0.075 (2)	0.0182 (15)	0.0457 (17)	0.0238 (17)
C10	0.0617 (18)	0.0580 (18)	0.075 (2)	0.0287 (15)	0.0436 (17)	0.0208 (16)
C11	0.0386 (13)	0.0403 (13)	0.0372 (13)	0.0119 (10)	0.0157 (11)	0.0063 (10)
C12	0.0373 (12)	0.0372 (12)	0.0346 (12)	0.0100 (10)	0.0112 (10)	0.0072 (10)
C13	0.0568 (16)	0.0407 (14)	0.0500 (16)	0.0176 (13)	0.0181 (13)	0.0071 (12)
C14	0.073 (2)	0.0354 (14)	0.074 (2)	0.0138 (14)	0.0228 (18)	0.0132 (14)
C15	0.078 (2)	0.0462 (17)	0.097 (3)	0.0088 (16)	0.049 (2)	0.0330 (17)
C16	0.067 (2)	0.0456 (16)	0.090 (2)	0.0122 (14)	0.0504 (19)	0.0229 (16)
C17	0.0393 (15)	0.0692 (19)	0.0479 (15)	0.0069 (13)	0.0247 (13)	0.0119 (14)
C18	0.0305 (12)	0.0452 (14)	0.0499 (15)	0.0083 (10)	0.0196 (11)	0.0111 (11)
C19	0.0434 (15)	0.0562 (17)	0.0664 (19)	−0.0021 (13)	0.0262 (14)	0.0210 (15)
C20	0.0389 (15)	0.0493 (16)	0.077 (2)	−0.0103 (12)	0.0170 (15)	0.0051 (15)
C21	0.0367 (13)	0.0525 (16)	0.0486 (16)	0.0028 (12)	0.0087 (12)	−0.0012 (13)
C22	0.0384 (13)	0.0467 (14)	0.0441 (14)	0.0028 (11)	0.0165 (12)	0.0102 (12)
C23	0.0314 (12)	0.0347 (12)	0.0455 (14)	0.0047 (10)	0.0160 (11)	0.0061 (10)
C24	0.0407 (14)	0.0352 (13)	0.0491 (15)	0.0008 (11)	0.0104 (12)	0.0079 (12)
Zn1	0.04219 (19)	0.03371 (17)	0.04103 (19)	0.00763 (12)	0.02487 (14)	0.00712 (12)
N1	0.0539 (16)	0.077 (2)	0.0604 (18)	−0.0016 (15)	0.0026 (14)	−0.0046 (15)
N2	0.0599 (15)	0.0532 (14)	0.0574 (15)	0.0075 (12)	0.0395 (13)	0.0110 (11)
N3	0.0587 (14)	0.0411 (12)	0.0493 (13)	0.0113 (10)	0.0346 (11)	0.0102 (10)
N4	0.0606 (15)	0.0407 (12)	0.0460 (13)	0.0110 (11)	0.0254 (11)	0.0056 (10)
N5	0.0450 (12)	0.0422 (12)	0.0544 (13)	0.0050 (10)	0.0288 (11)	0.0124 (10)
N6	0.0392 (11)	0.0351 (10)	0.0414 (11)	0.0058 (9)	0.0202 (9)	0.0059 (9)
N7	0.0463 (12)	0.0344 (11)	0.0526 (13)	0.0084 (9)	0.0252 (11)	0.0104 (9)
O1	0.0434 (10)	0.0361 (9)	0.0475 (10)	0.0010 (7)	0.0204 (8)	0.0079 (8)
O2	0.0457 (15)	0.393 (7)	0.0555 (17)	0.021 (3)	0.0304 (13)	0.017 (3)
O3	0.0376 (10)	0.0464 (10)	0.0458 (10)	0.0068 (8)	0.0176 (8)	0.0030 (8)
O4	0.0814 (15)	0.0387 (10)	0.0495 (12)	−0.0046 (10)	0.0213 (11)	0.0036 (9)
O5	0.0723 (15)	0.0643 (14)	0.0923 (18)	−0.0264 (12)	0.0529 (15)	−0.0085 (13)
O6	0.0778 (18)	0.140 (3)	0.0511 (14)	−0.0108 (18)	0.0169 (14)	0.0024 (16)
O7	0.124 (3)	0.145 (3)	0.082 (2)	−0.076 (3)	−0.021 (2)	0.003 (2)
O1W	0.196 (7)	0.229 (8)	0.280 (10)	0.104 (7)	0.185 (8)	0.091 (8)

### Geometric parameters (Å, °)

**Table:**

C1—N2	1.343 (4)	C16—N7	1.333 (4)
C1—C2	1.357 (5)	C16—H16	0.9300
C1—H1	0.9300	C17—O2	1.221 (4)
C2—C3	1.396 (4)	C17—O3	1.231 (3)
C2—H2	0.9300	C17—C18	1.506 (4)
C3—N3	1.334 (4)	C18—C19	1.390 (4)
C3—C4	1.460 (4)	C18—C23	1.396 (4)
C4—N4	1.352 (4)	C19—C20	1.374 (5)
C4—C5	1.390 (4)	C19—H19	0.9300
C5—C6	1.369 (5)	C20—C21	1.371 (5)
C5—H5	0.9300	C20—H20	0.9300
C6—C7	1.390 (6)	C21—C22	1.376 (4)
C6—H6	0.9300	C21—N1	1.474 (4)
C7—C8	1.382 (5)	C22—C23	1.383 (4)
C7—H7	0.9300	C22—H22	0.9300
C8—N4	1.330 (4)	C23—C24	1.518 (3)
C8—H8	0.9300	C24—O5	1.235 (4)
C9—N5	1.341 (4)	C24—O4	1.250 (4)
C9—C10	1.363 (5)	Zn1—O1	2.0871 (18)
C9—H9	0.9300	Zn1—N3	2.089 (2)
C10—C11	1.393 (4)	Zn1—O3	2.0981 (18)
C10—H10	0.9300	Zn1—N6	2.147 (2)
C11—N6	1.336 (3)	Zn1—N7	2.188 (2)
C11—C12	1.460 (4)	Zn1—N4	2.287 (2)
C12—N7	1.342 (3)	N1—O7	1.204 (4)
C12—C13	1.391 (4)	N1—O6	1.211 (4)
C13—C14	1.371 (5)	N2—N3	1.341 (3)
C13—H13	0.9300	N2—H2A	0.8600
C14—C15	1.359 (5)	N5—N6	1.337 (3)
C14—H14	0.9300	N5—H5A	0.8600
C15—C16	1.379 (4)	O1—H1W	0.820 (13)
C15—H15	0.9300	O1—H2W	0.82 (3)
			
N2—C1—C2	108.1 (3)	C18—C19—H19	119.3
N2—C1—H1	126.0	C21—C20—C19	118.3 (3)
C2—C1—H1	126.0	C21—C20—H20	120.9
C1—C2—C3	105.1 (3)	C19—C20—H20	120.9
C1—C2—H2	127.4	C20—C21—C22	122.1 (3)
C3—C2—H2	127.4	C20—C21—N1	118.7 (3)
N3—C3—C2	109.9 (3)	C22—C21—N1	119.2 (3)
N3—C3—C4	116.7 (2)	C21—C22—C23	119.5 (3)
C2—C3—C4	133.4 (3)	C21—C22—H22	120.2
N4—C4—C5	122.4 (3)	C23—C22—H22	120.2
N4—C4—C3	114.5 (2)	C22—C23—C18	119.3 (2)
C5—C4—C3	123.1 (3)	C22—C23—C24	117.3 (2)
C6—C5—C4	118.8 (3)	C18—C23—C24	123.3 (2)
C6—C5—H5	120.6	O5—C24—O4	125.7 (3)
C4—C5—H5	120.6	O5—C24—C23	116.6 (2)
C5—C6—C7	119.3 (3)	O4—C24—C23	117.6 (3)
C5—C6—H6	120.4	O1—Zn1—N3	96.46 (8)
C7—C6—H6	120.4	O1—Zn1—O3	86.38 (7)
C8—C7—C6	118.5 (4)	N3—Zn1—O3	96.57 (9)
C8—C7—H7	120.8	O1—Zn1—N6	94.67 (8)
C6—C7—H7	120.8	N3—Zn1—N6	163.63 (9)
N4—C8—C7	123.2 (3)	O3—Zn1—N6	96.08 (7)
N4—C8—H8	118.4	O1—Zn1—N7	168.95 (8)
C7—C8—H8	118.4	N3—Zn1—N7	94.18 (9)
N5—C9—C10	107.6 (3)	O3—Zn1—N7	89.49 (8)
N5—C9—H9	126.2	N6—Zn1—N7	75.57 (8)
C10—C9—H9	126.2	O1—Zn1—N4	93.00 (8)
C9—C10—C11	105.1 (3)	N3—Zn1—N4	73.48 (9)
C9—C10—H10	127.5	O3—Zn1—N4	169.91 (8)
C11—C10—H10	127.5	N6—Zn1—N4	94.01 (8)
N6—C11—C10	110.3 (2)	N7—Zn1—N4	92.85 (8)
N6—C11—C12	118.0 (2)	O7—N1—O6	122.6 (3)
C10—C11—C12	131.8 (2)	O7—N1—C21	118.0 (4)
N7—C12—C13	121.8 (3)	O6—N1—C21	119.4 (3)
N7—C12—C11	114.8 (2)	N3—N2—C1	110.5 (3)
C13—C12—C11	123.4 (2)	N3—N2—H2A	124.7
C14—C13—C12	118.9 (3)	C1—N2—H2A	124.7
C14—C13—H13	120.6	C3—N3—N2	106.3 (2)
C12—C13—H13	120.6	C3—N3—Zn1	120.6 (2)
C15—C14—C13	119.5 (3)	N2—N3—Zn1	132.65 (19)
C15—C14—H14	120.3	C8—N4—C4	117.9 (3)
C13—C14—H14	120.3	C8—N4—Zn1	127.8 (2)
C14—C15—C16	119.0 (3)	C4—N4—Zn1	114.3 (2)
C14—C15—H15	120.5	N6—N5—C9	111.4 (2)
C16—C15—H15	120.5	N6—N5—H5A	124.3
N7—C16—C15	122.8 (3)	C9—N5—H5A	124.3
N7—C16—H16	118.6	C11—N6—N5	105.7 (2)
C15—C16—H16	118.6	C11—N6—Zn1	115.52 (17)
O2—C17—O3	126.1 (3)	N5—N6—Zn1	138.68 (16)
O2—C17—C18	116.8 (3)	C16—N7—C12	118.1 (2)
O3—C17—C18	117.0 (2)	C16—N7—Zn1	126.03 (19)
C19—C18—C23	119.3 (3)	C12—N7—Zn1	115.32 (17)
C19—C18—C17	117.7 (3)	Zn1—O1—H1W	101 (3)
C23—C18—C17	122.8 (2)	Zn1—O1—H2W	119 (3)
C20—C19—C18	121.4 (3)	H1W—O1—H2W	114 (3)
C20—C19—H19	119.3	C17—O3—Zn1	139.12 (19)

### Hydrogen-bond geometry (Å, °)

**Table:**

*D*—H···*A*	*D*—H	H···*A*	*D*···*A*	*D*—H···*A*
O1—H2W···O5^i^	0.82 (3)	1.81 (3)	2.627 (3)	174 (3)
N5—H5A···O4^i^	0.86	1.95	2.788 (3)	166
N2—H2A···O2	0.86	1.82	2.606 (4)	150

Symmetry codes: (i) −*x*+2, −*y*+2, −*z*+1.
